# Antimicrobial activity, acute toxicity and cytoprotective effect of Crassocephalum vitellinum (Benth.) S. Moore extract in a rat ethanol-HCl gastric ulcer model

**DOI:** 10.1186/1756-0500-7-91

**Published:** 2014-02-19

**Authors:** Mainen J Moshi, Ramadhani SO Nondo, Emmanuel E Haule, Rogasian LA Mahunnah, Abdul W Kidukuli

**Affiliations:** 1 Department of Biological and Preclinical Studies, Muhimbili University of Health and Allied Sciences, P.O. Box 65001, Dar es Salaam, Tanzania; 2 Department of Medical Botany, Plant Breeding and Agronomy, Institute of Traditional Medicine, Muhimbili University of Health and Allied Sciences, P.O. Box 65001, Dar es Salaam, Tanzania

**Keywords:** *Crassocephalum vitellinum*, Traditional medicine, Gastric cytoprotection, Antimicrobial, Acute, Toxicity

## Abstract

**Background:**

A decoction of *Crassocephallum vitellinum* (Benth.) S. Moore (Asteraceae) is used in Kagera Region to treat peptic ulcers. This study seeks to evaluate an aqueous ethanol extract of aerial parts of the plant for safety and efficacy.

**Methods:**

An 80% ethanolic extract of *C. vitellinum* at doses of 100, 200, 400 and 800 mg/kg body wt was evaluated for ability to protect Sprague Dawley rats from acidified ethanol gastric ulceration in comparison with 40 mg/kg body wt pantoprazole. The extract and its dichloromethane, ethyl acetate, and aqueous fractions were also evaluated for acute toxicity in mice, brine shrimp toxicity, and antibacterial activity against four Gram negative bacteria; *Escherichia coli* (ATCC 25922), *Salmonella typhi* (NCTC 8385), *Vibrio cholera* (clinical isolate), and *Streptococcus faecalis* (clinical isolate). The groups of phytochemicals present in the extract were also determined.

**Results:**

The ethanolic extract of *C. vitellinum* dose-dependently protected rat gastric mucosa against ethanol/HCl insult to a maximum of 88.3% at 800 mg/kg body wt, affording the same level of protection as by 40 mg/kg body wt pantoprazole. The extract also exhibited weak antibacterial activity against *S. typhi* and *E. coli*, while its ethyl acetate, dichloromethane and aqueous fractions showed weak activity against *K. pneumonia, S.typhi, E. coli* and *V. cholera*. The extract was non-toxic to mice up to 5000 mg/kg body wt, and the total extract (LC_50_ = 37.49 μg/ml) and the aqueous (LC_50_ = 87.92 μg/ml), ethyl acetate (LC_50_ = 119.45 μg/ml) and dichloromethane fractions (88.79 μg/ml) showed low toxicity against brine shrimps. Phytochemical screening showed that the extract contains tannins, saponins, flavonoids, and terpenoids.

**Conclusion:**

The results support the claims by traditional healers that a decoction of *C.vitellinum* has antiulcer activity. The mechanism of cytoprotection is yet to be determined but the phenolic compounds present in the extract may contribute to its protective actions. However, the dose conferring gastro-protection in the rat is too big to be translated to clinical application; thus bioassay guided fractionation to identify active compound/s or fractions is needed, and use of more peptic ulcer models to determine the mechanism for the protective action.

## Background

The Kagera region has a culture rich in traditional medicine practice, but the majority of plants used in this region are poorly documented and have not yet been evaluated for safety and efficacy. *Crassocephallum vitellinum* (Benth.) S. Moore (ASTERACEAE); Synonym: *Gynura vitellina* Benth is among plants used in Bukoba for the treatment of peptic ulcer disease [1]. The aerial parts of *C. vitellinum* are boiled with water and the resulting decoction is taken regularly for management of peptic ulcers [1]. Reports from other areas show that in Kenya the plant is used for the treatment of stomach complications, malaria and mouth infections in children [2], while in Zaire the leaves are burnt with those of the cultivated yam and the ash is applied to scarifications for the treatment of swollen legs [3]. In Southern Rwanda *C.vitellinum* is used as a hepatoprotective remedy which has eventually been linked to its antioxidant properties [4,5]. An extract of the plant protected rats against acetaminophen induced liver toxicity, and in addition it reversed barbiturate induced sleep in guinea pigs [4]. The leaves are used for the treatment of female sterility, strong fever, dysmenorrhea, constipation, childhood diseases and facilitate the deliverance of placenta [4]. The filtrate of crushed leaves and flowers of the plant combined with those of *Plantago palmata* Hoof.f., *Vernonia auriculifera* Hiern and *Physalis peruviana* L. is used for treatment of whooping cough [6], while the leaves are used for management of anorexia [7] and for antenatal care [8]. The leaves are used in veterinary medicine for treatment of mastitis, fever, and to facilitate lactation and activity has been linked to the flavonoid content of the leaves [9]. The antioxidant and antibacterial activity of the essential oils of the aerial parts of the plant was recently reported [10]. In refugee camps in Tanzania, traditional healers use the leaves of *C. vitellinum* for constipation and abortion [11].The leaves are also used by the Haya people to treat gonorrhea [1].

This study was done to evaluate the efficacy of an extract of the aerial parts for protection against acidified ethanol induced gastric ulceration in Spague Dawley rats. The extract was also tested for antibacterial activity. Phytochemical screening was done to determine the groups of compounds present in the extract, while acute toxicity in mice and brine shrimp lethality test were done to determine safety of the extract.

## Methods

### Materials

Tryptone Soya broth was purchased from HIMEDIA® (Himedia Laboratories Pvt Ltd, Mumbai, INDIA; *p-*Iodonitrotetrazolium chloride (INT) from SIGMA® (Sigma- Aldrich®, St Louis, USA); pantoprazole (lyophilized powder for i.v injection; Batch No. JKJ 3534D and manufactured by Sun Pharmaceutical Industries Ltd, Halol-Baroda Highway, Halol-389350, Gujarat, India.), was sourced from a local pharmacy. Ethanol (absolute) was purchased from Fluka Chemie GmbH (Sigma-Aldrich®, Zwijndrecht, Netherlands), while dimethyl sulfoxide (DMSO) was purchased from Sigma® (Poole, Dorset, UK). Cyclophosphamide, NEOPHOS 500® (CIPLA Ltd, MIDC Boisar, INDIA) was purchased from a local Pharmacy in Dar es Salaam, Tanzania. *Escherichia coli* (ATCC 25922), *Salmonella typhi* (NCTC 8385), *Vibrio cholerae* (clinical isolate), and *Klebsiella pneumoniae* (clinical isolate) were obtained from the Department of Microbiology and Immunology, Muhimbili University of Health and Allied Sciences (MUHAS). The brine shrimp eggs were bought from Aquaculture Innovations (Grahamstown 6140, South Africa) and sea salt was prepared locally by evaporating water collected from the Indian Ocean, along the Dar es Salaam Coast.

### Collection of plant material

The aerial parts of *Crassocephallum vitellinum* (Benth.) S. Moore (ASTERACEAE) were collected by an experienced botanist, Mr. Haji O. Selemani of the Department of Botany, University of Dar es Salaam and voucher specimen no. MH 164569 is kept at the Herbarium of the Institute of Traditional Medicines, Muhimbili University of Health and Allied Sciences.

### Preparation of plant extracts

The air-dried plant material was ground into powder using a plant milling machine. The plant material was macerated in 80% ethanol at room temperature for 24 h and filtered through Whatman No.1 filter paper. This procedure was repeated 3 times to ensure complete extraction of plant material. The pooled extract was concentrated by evaporation under reduced pressure in a rotary evaporator at 40 ºC. The extract yield was 31 g from 292 g of dry plant material (10.62%).

### Testing for antiulcer activity

Both male and female Sprague Dawley rats weighing 100-188 g were used. The rats were fasted for 36 h but allowed free access to drinking water. Thirty rats were randomly allocated to 5 groups of 6 rats each. The rats in group one (Solvent control) were pre-dosed orally with 5 ml/kg body wt of 1% tween 80 in normal saline; rats in group two were pre-dosed orally with 40 mg/kg body wt pantoprazole (Positive control), and rats in groups 3, 4 and 5 were pre-dosed orally with 200, 400 and 800 mg/kg body wt solution of *C. vitellinum* extract one hour before oral administration of 5 ml/kg body wt of the ulcerogenic mixture. The ulcerogenic mixture contained 80% ethanol, 5% hydrochloric acid and 15% distilled water. The animals were euthanized by ether anaesthesia 4 h after administration of acidified ethanol. The stomachs were removed, cut open along the greater curvature, washed with normal saline and observed for the severity of ulcers. The degree of ulceration (Ulcer index) was graded according to a method previously described by Shay and colleagues [12] as follows: 0 = no lesions (normal stomach); 0.5 = hyperemia (red coloration); 1 = hemorrhagic spots, 2 = 1–5 small ulcers; 3 = many small ulcers, 4 = many small and large ulcers; 6 = stomach full of ulcers along with perforations. Percentage protection was calculated by comparison with the untreated control group Percentage protection [12]

$$=100\;-\;\frac{\mathrm{Mean}\;\mathrm{ulcer}\;\mathrm{index}\;\mathrm{of}\;\mathrm{treated}\;\mathrm{group}}{\mathrm{Mean}\;\mathrm{ulcer}\;\mathrm{index}\;\mathrm{of}\;\mathrm{control}\;\mathrm{group}}\times \;100$$

### Statistical analysis

The results are expressed as mean ± standard deviation (SD). The results of mean ulcer index were compared using the non-parametric Kruskal-Wallis test.

### Testing for antibacterial activity

#### Test organisms

Four Gram-negative bacteria (enterobactericeae) namely *Escherichia coli, Salmonella typhi, Vibro cholera,* and *Klebsiella pneumonea* were used, which were obtained from the Department of Microbiology and Immunology, Muhimbili University of Health and Allied Sciences.

#### Determination of minimum inhibitory concentrations (MICs)

Minimum inhibitory concentrations (MICs) were determined using the microdilution method [13]. A stock solution of the extract was prepared by dissolving 100 mg of the 80% ethanol extract into 1 ml of DMSO (100 mg/ml). Each of the 96 well microtitre plates were first preloaded with 100 μl of tryptic soya broth followed by addition of 100 μl of the extract into the first wells of the rows to make a total volume of 200 μl in the first wells. After thorough mixing 100 μl were transferred from the first row wells into the next row wells. The process was repeated sequentially to the last well at the bottom where 100 μl was discarded. Thereafter, 100 μl of the bacterial suspension (0.5Mac Farland standard turbidity) was then added to each well to make a final volume of 200 μl in each well. Gentamicin sulphate (100 μg/ml) was used as a standard drug. Rows containing broth, DMSO and bacteria were included as negative controls (solvent control) and rows containing broth and bacteria only were included in order to see whether there was bacterial growth or not (growth control). The plates were then incubated at 37°C for 24 h. After incubation for 24 h, at 37°C, 40 μl of 0.02% p-iodonitrotetrazolium (INT) chloride solution was added to each well followed by incubation for 1 h at 37°C. Bacterial growth was indicated by a change in color to pink in the wells. Absence of bacterial growth was indicated by no color change of the dye. The first concentration at which no bacterial growth occurred was taken as the MIC.

#### Brine shrimps lethality test

The brine shrimp lethality test (BST) was used to predict the presence, in the extract, of cytotoxic activity [14]. Solutions of the extract were made in DMSO at concentrations ranging from 8–240 μg/ml and incubated in duplicate vials with brine shrimp larvae. Ten brine shrimp larvae were placed in each of the duplicate vials. Control brine shrimp larvae were placed in a mixture of artificial sea water (3.8 g/l sea salt) and DMSO only. After 24 h the nauplii were examined against a lighted background, and the average number of live larvae in each duplicate vial was determined. The mean percentage mortality was plotted against the logarithm of concentrations and the concentration killing fifty percent of the larvae (LC_50_) was determined from the graph [15].

### Data analysis

The mean results of brine shrimp mortality against the logarithms of concentrations were plotted using the Fig P computer program (Biosoft Inc, USA), which also gives the regression equations. The regression equations were used to calculate LC_16_, LC_50_ and LC_84_ values. Confidence intervals (95% CI) were then calculated using the three results [14,15]. An LC_50_ value greater than 100 μg/ml was considered to represent an inactive compound or extract.

#### Acute toxicity test

Acute toxicity was done according to OECD guidelines 425 [16]. Both male and female Theiller’s original albino mice were used. The mice were acclimatized in an air conditioned room at 20°C for 7 days before experimentation. Before dosing with extracts the mice were starved for 18–24 h with access to adequate drinking water, in cages with wire mesh bottoms to prevent coprophagy. Initially a dose of 1000 mg/kg body wt was administered to a group of 6 mice (3 male and 3 female), and mice observed for signs of immediate toxicity and/or death for 72 h. If no toxicity was observed another group of 3 male and 3 female mice was given a dose of 2000 mg/kg body wt and the same observations made. If no signs of toxicity or death occurred doses were sequentially increased to 3000, 4000, and 5000 mg/kg body wt, respectively. Extracts were solubilized in 1% tween 80 in normal saline and administered at a single dose volume of 5 ml/kg body wt or two doses of 5 ml/kg body wt depending on solubility, within a one hour interval. A control group was administered a single 5 ml or 5 ml/kg body wt of 1% tween 80 twice to match with the dose of administered plant extract.

#### Phytochemical screening

The 80% ethanol extract was tested for the presence of steroids, saponins, flavonoids, terpenoids, cardiac glycosides, alkaloids and tannins using standard methods [17,18].

#### Ethical clearance

This study was given ethical clearance by the Muhimbili University of Health and Allied Sciences Institutional Review Board.

## Results

### Antiulcer activity

**Figure 1:**
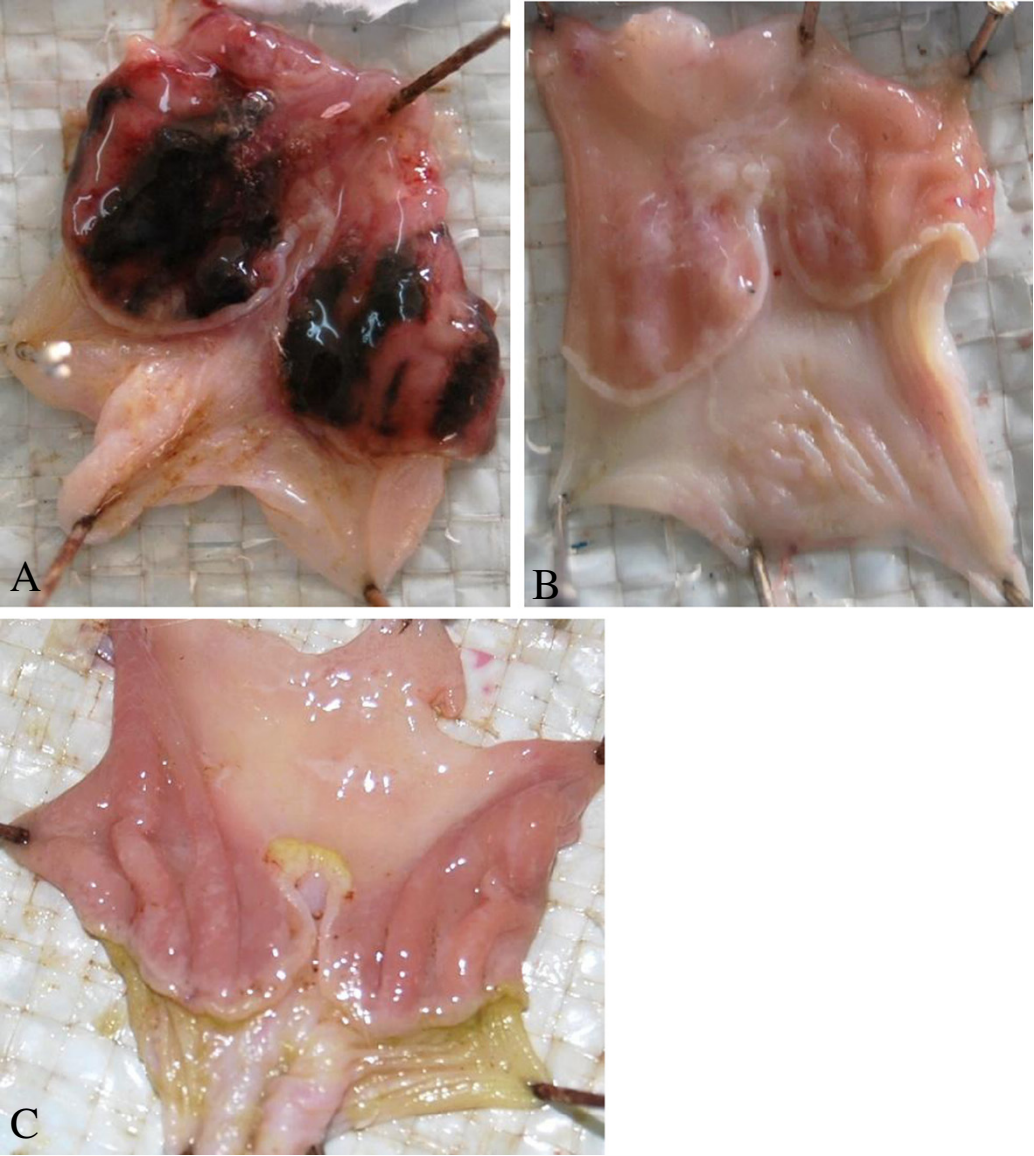
**Photograph showing the stomach following insult with ethanol/HCl (80%/5%) mixture.** In **(A)** the rat was pre-dosed with the solvent before insult with ethanol/HCl mixture. In **(B)** the rat was pre-dosed with 800 mg/kg body wt of 80% ethanol extract of *C. vitellinum* before exposure to the ulcerative mixture and in **(C)** the rat was pre-dosed with 40 mg/kg body wt pantoprazole.

**Figure 2:**
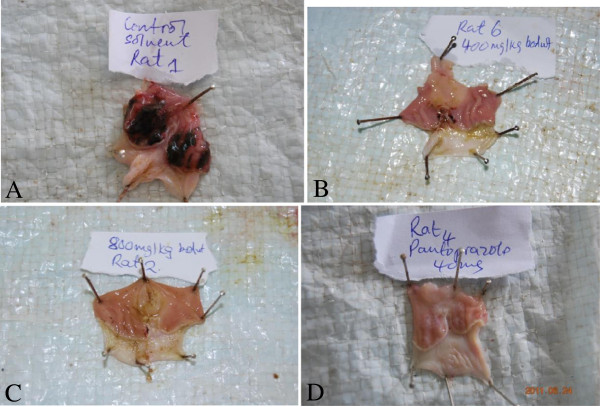
**Photographs of rat gastric mucosa showing the dose-dependent effect of *C. vitellinum *against acidified ethanol ulceration of rat gastric mucosa. A** = Solvent control; **B** = 400 mg/kg body wt plant extract; **C** = 800 mg/kg body wt plant extract; **D** = 40 mg/kg body wt pantoprazole.

The 80% ethanol extract of *C. vitellinum* showed a dose-dependent protection against ethanol/HCl induced gastric ulceration. Table 1 shows that the extract protected the rat stomach from ulceration by 55% at a dose of 200 mg/kg body wt (P ≤ 0.001) and this increased to 71.7(P ≤ 0.001), and 88.3%(P ≤ 0.001) at 400 and 800 mg/kg body wt as compared to solvent treated rats, respectively. This was the same percentage protection as 40 mg/kg body wt of the proton pump inhibitor, pantoprazole. Figure 1 shows that at 800 mg/kg body wt the stomach appeared similar to that of the rats pretreated with 40 mg/kg body wt pantoprazole. Figure 2 shows the dose–response relationship for control, 400 and 800 mg/kg body wt of the plant extract

**Table 1:** **Antiulcer activity of an 80% ethanol extract of aerial parts of *C. vitellinum***

**Group No.**	**Dose of extract (mg/kg body wt)**	**Mean ulcer index (n = 6)**	**% age protection**
**1**	Solvent control	6	0
**2**	200	2.7 ± 1.0	55.0*
**3**	400	1.7 ± 1.4	71.7*
**4**	800	0.7 ± 0.4	88.3*
**5.**	40 mg/kg body wt pantoprazole	0.7 ± 0.9	88.3*

Each value is a mean of results of 6 rats. * = significant P ≤ 0.001 compared to solvent controls.

### Antibacterial activity

The results for antibacterial activity (Table 2) indicate that the total extract had weak activity against *Salmonella typhi* and *Escherichia coli* with MIC of 3.125 mg/ml. When the extract was fractionated to aqueous, ethyl acetate and dichloromethane fractions the results show that the ethyl acetate fraction was active against the four bacteria used; i.e. *K. pneumoniae, S. typhi, E. Coli* and *V. cholera*, and was active against more bacteria than the total extract. Both the dichloromethane and ethyl acetate extracts had very weak activity against all the four bacteria used.

**Table 2:** **Antibacterial activity of ethyl acetate, DCM and aqeous fractions of *C.vitellinum *** **ethanol extract**

**Gram-ve test isolates**	**MICs (mg/ml) of 80%ethanol extract and its fractions**			
	**80% ethanol extract (total)**	**Ethyl acetate (fraction)**	**DCM (fraction)**	**Aqeous (fraction)**
*K.pneumonea*	**-**	**6.25**	**12.5**	**12.5**
*S.typhii*	3.125	**3.125**	**12.5**	**12.5**
*E.coli*	3.125	**3.125**	**6.25**	**6.25**
*V.cholera*	**-**	**3.125**	**12.5**	**12.5**

### Brine shrimp lethality test

The brine shrimp test results (Table 3) show that generally the total extract (LC_50_ = 37.49 μg/ml) and the aqueous, dichloromethane and ethyl acetate fractions all exhibited moderately low brine shrimp toxicity.

**Table 3:** **The effect *C. vitellinum *** **total extract and its fractions on brine shrimp toxicity**

** Extracts**	**LC**_**50 **_**(μg/ml)**	**95% CI(μg/ml)**
80% ethanol extract	37.49	27.98 - 50.16
Aqueous fraction	87.92	63.71-121.33
Ethyl acetate fraction	119.45	86.56-164.84
Dichloromethane fraction	88.79	69.37-113.65

### Acute toxicity in mice

The total extract up to 5000 mg/kg body wt did not show any signs of toxicity to mice and all the mice survived to the 28 days of observation period.

### Phytochemical Screening

The results of phytochemical screening show that the 80% ethanol extract of *Crassocephalum vitellinum* contains tannins, saponins, flavonoids and triterpenes.

## Discussion

The mechanism of ethanol gastric ulceration is due to direct toxic effect on the epithelium leading to necrotic lesions, depletion of gastric mucus which breaks the mucosal barrier, increase permeability of gastric mucosa leading to increased leakage of hydrogen ions from the lumen, back diffusion of acid, and decrease transluminal electrical potential difference [19-21]. Ethanol also causes changes in mucosal blood flow, destroy microvascular and non-vascular cells, mast cell degranulation, neutophil mediated mucosal injury, and depletion of certain oxygen free radical scavengers [22]. In addition to depletion of free radical scavengers, free radicals are released from the metabolism of ethanol [22], and this if further exacerbated by inhibition of the biosynthesis of cytoprotective prostaglandins [23].

In this study pantoprazole showed a clear protective action against acidified ethanol ulceration, probably suggesting the role of gastric acid in the acidified ethanol-induced gastric ulceration. Appearance of the pantoprazole treated rat gastric mucosa shows a big difference in gross appearance as compared to the solvent controls. On the other hand the aqueous ethanol extract of *C. vitellinum,* at 800 mg/kg body wt, conferred the same level of protection to the rat gastric mucosa (88.3%) as 40 mg/kg body wt pantoprazole. An important question here is what is the basis for the protective action of the extract. Certainly there is no adequate evidence to allow us to suggest the mechanism of gastro-protection. The results of phytochemical screening showed that the extract of the aerial parts of *C. vitellinum* contains tannins, flavonoids and terpenoids which are phenolic compounds. Plant phenolic compounds are known to be antioxidants and free radical scavengers [24]. Owing to the established fact that ethanol-induced gastric ulceration is associated with neutrophil mediated mucosal injury through the release of oxygen free radicals, proteases and lysosomal enzymes [22], there is a strong indication that the *C. vitellinum* extract owes its anti-ulcerogenic effect partly due to the presence of flavonoids, tannins and terpenoids which have antioxidant and free radical scavenging activity. The anti-oxidant properties of this plant have been reported in another study [4].

The 80% ethanol extract and its fractions exhibited some antibacterial activity against four Gram negative bacteria, which seems to be highest in the ethyl acetate fractions. It was intended to test the extracts for inhibitory effect against *Helicobacter pylori* which is implicated in peptic ulcer disease. However, it was not possible to get these organisms from any commercial source or through other laboratories. It was decided to test the extracts against the four enterobacteria to just get indicative results, but it does not mean in any way that getting positive results is a direct indication that the extracts would also automatically be active against *H. pylori* but does suggest that the extracts may be useful for treating other intestinal conditions related to infection by these bacteria. This notion is supported by some of the traditional medicine uses [5,8,9].

The extract of *C. vitellinum* showed no significant changes in animal behavior or adverse signs of toxicity during post treatment evaluation of the mice following treatment with up to 5000 mg/kg body wt, thus suggesting that it is safe when administered acutely. Similar findings were observed in brine shrimp toxicity test in which both the total extract and its fractions showed minimal toxicity.

## Conclusion

These results support the claim by traditional healers that the extract of the aerial parts of *C.vitellinum* has antiulcer activity. Since the dose conferring maximum protection is very big it is unlikely that the results can have immediate clinical application. While it may be suggested that phenolic compounds contribute to the protective actions of the extract, there is need to do bioassay guided fractionation to identify active compound/s or fractions. The mechanism for the protective action also remains to be a subject for further studies.

## Abbreviations

BST: Brine shrimp tests; CI: Confidence interval; DMSO: Dimethylsulfoxide; HCl: Hydrochloric acid; INT: p-iodonitrotetrazolium; LC: Lethal concentration; MIC: Minimum inhibitory concentration; OECD: Organization for economic co-operation and development.

## Competing interests

The authors have no competing interests for this research, and share the aspirations of the traditional healers of the Kagera Region to improve healthcare services in their community.

## Authors’ contributions

MJM did the ethnomedical studies that documented these plants and identified the plants for this study; participated in advising design of the study, provided support and guidance in the laboratory and provided the theoretical guidance of the project. He also drafted this manuscript. RSON participated in the experiments and was the laboratory supervisor for the study. He also participated in designing the study. EEH did some of the experiments, including preparation of the plant material, extraction, bioassays etc. under the guidance of RSON who worked closely with him in the laboratory. RLAM is a co-mentor of EEH and participated in advising design of the study and proposal development. AWK did phytochemical screening experiments and brine shrimp lethality experiments. All the authors read and approved the manuscript.
